# Defining the role of neutrophils in the lung during infection: Implications for tuberculosis disease

**DOI:** 10.3389/fimmu.2022.984293

**Published:** 2022-09-20

**Authors:** E. Gaffney, D. Murphy, A. Walsh, S. Connolly, S. A. Basdeo, J. Keane, J. J. Phelan

**Affiliations:** Department of Clinical Medicine, Trinity Translational Medicine Institute, Trinity Centre for Health Sciences, St. James’s Hospital, Dublin, Ireland

**Keywords:** metabolism, tuberculosis, immunology, neutrophils, cell interactions, granuloma, neutrophil (PMN), granulocyte

## Abstract

Neutrophils are implicated in the pathogenesis of many diseases involving inflammation. Neutrophils are also critical to host defence and have a key role in the innate immune response to infection. Despite their efficiencies against a wide range of pathogens however, their ability to contain and combat *Mycobacterium tuberculosis* (Mtb) in the lung remains uncertain and contentious. The host response to Mtb infection is very complex, involving the secretion of various cytokines and chemokines from a wide variety of immune cells, including neutrophils, macrophages, monocytes, T cells, B cells, NK cells and dendritic cells. Considering the contributing role neutrophils play in the advancement of many diseases, understanding how an inflammatory microenvironment affects neutrophils, and how neutrophils interact with other immune cells, particularly in the context of the infected lung, may aid the design of immunomodulatory therapies. In the current review, we provide a brief overview of the mechanisms that underpin pathogen clearance by neutrophils and discuss their role in the context of Mtb and non-Mtb infection. Next, we examine the current evidence demonstrating how neutrophils interact with a range of human and non-human immune cells and how these interactions can differentially prime, activate and alter a repertoire of neutrophil effector functions. Furthermore, we discuss the metabolic pathways employed by neutrophils in modulating their response to activation, pathogen stimulation and infection. To conclude, we highlight knowledge gaps in the field and discuss plausible novel drug treatments that target host neutrophil metabolism and function which could hold therapeutic potential for people suffering from respiratory infections.

## Introduction

Neutrophils are the most abundant leukocyte population in humans, comprising of about 50-70% of all leukocytes in the circulation (1). In the absence of stimulation, neutrophils are short lived cells; circulating for approximately 8 to 20 hours before undergoing apoptosis (2–4). Neutrophils provide a crucial first line of defence against invading pathogens, such as *Mycobacterium tuberculosis* (Mtb). The production of cytokines and chemokines by other immune cells recruits neutrophils to sites of infection in a process called chemotaxis and helps draw them deep into inflamed tissues (5). Upon activation, neutrophils use an arsenal of effector mechanisms to kill invading pathogens. They also produce proinflammatory cytokines and chemokines which aid in recruiting additional immune cells to the site of infection (6). Various immune cells, including macrophages, monocytes, T cells, B cells, NK cells, and dendritic cells, can significantly influence bystander neutrophil recruitment, activation, and function, both through direct cell-to-cell contact and *via* the production of soluble factors, such as cytokines and chemokines.

As neutrophils are such an abundant component of the innate immune system, their highly inflammatory actions have potentially severe consequences for host tissues. Thus, neutrophils must be tightly regulated to resolve inflammation and limit tissue damage. If the regulatory mechanisms controlling the clearance of apoptotic neutrophils are impaired or the infection in question cannot be resolved, the actions of neutrophils can become deleterious to the host (7). Indeed, the pathogenesis of many lung diseases involves neutrophilic inflammation, where significant neutrophil recruitment contributes to tissue injury (8).

**Figure 1:**
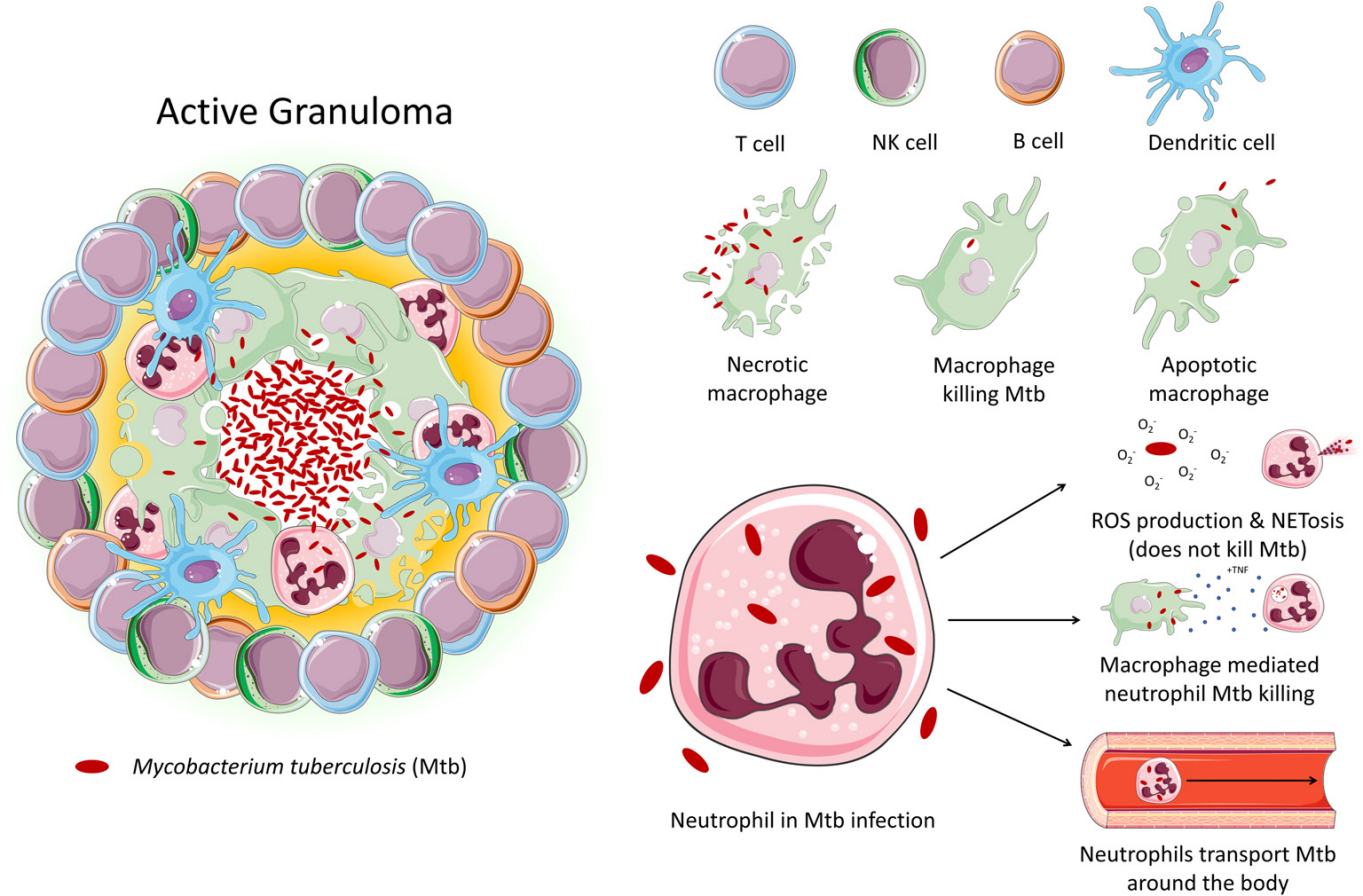
An active granuloma. This mass of cells represents an area of inflammation upon infection with Mtb. Immune cells crowd around the bacteria to contain it. The neutrophil's role in tuberculosis is largely unexplored. Studies demonstrate that while neutrophil effector function is triggered by Mtb, neutrophils are unable to kill the bacteria. Instead, neutrophils may act as a ‘Trojan horse’, transporting the bacteria around the body. Macrophages have been shown to be able to induce neutrophil Mtb killing *via* phagocytosis.

Tuberculosis (TB) is an infectious disease caused by Mtb. Approximately 10 million people contracted TB in 2020, and 1.5 million people died from the disease, making TB the leading cause of death from a single infectious agent, after COVID-19. TB primarily affects the lungs, where it encounters various cell types, particularly alveolar macrophages and neutrophils, the main phagocytes that host the bacilli. Pulmonary inflammation due to interaction of Mtb with macrophages and other immune cells results in the recruitment of monocytes, neutrophils, and primed T cells and B cells to lungs, culminating in formation of a granuloma (**Figure 1**) (9). Granuloma formation has a dual role in both bacterial containment and persistence – while it aids the host by reducing the spread of Mtb throughout the body, it also facilitates bacterial survival and replication, resulting in latent TB infection, which can persist for years within the host without progressing to active TB disease (10).

The involvement of neutrophils in host defence in TB is controversial as they have been shown to have opposing roles, eliciting both protective and pathological effects in the host (11, 12). Given this conflicting role in the context of TB, host-directed therapies (HDTs) targeting neutrophils have become a recent topic of interest in the effort to improve TB treatment, especially for the treatment of other infectious agents, such as SARS-CoV-2 (13, 14). To develop effective HDTs, however, understanding neutrophils function in the inflamed human lung is vital. In this review we primarily describe the role of neutrophils in the context of Mtb infection. Step-by-step, we explore the influence of several immune cells on the priming, activation, and function of neutrophils and discuss if the immunometabolism of neutrophils during inflammation holds some promise for the development of novel neutrophil specific HDTs.

## An overview of the mechanisms underpinning pathogen clearance by neutrophils

Neutrophils are the first immune cells to arrive at the site of infection and are the predominant infected phagocytic cells in patients with active TB; however, their role in pathogenicity versus protection is unclear (15–17). Neutrophils act as a double-edged sword during Mtb infection; they are thought to be protective during early infection, but more pathogenic in late disease where neutrophilia is associated with lung damage and more severe disease (11, 12). Additionally, in the absence of intracellular killing, neutrophils can act as a “Trojan horse” which transports Mtb to various sites around the body, leading to systemic infection (15–17). In chronic TB disease, neutrophilia arises due to uncontrolled inflammation and dysfunctional neutrophils with impaired bactericidal activity, which further recruit and activate more pro-inflammatory neutrophils (15).

Neutrophils are equipped with an impressive array of anti-microbial functions to enable them to eliminate invading pathogens. These include phagocytosis, antibody-dependent cellular cytotoxicity (ADCC), neutrophil extracellular traps (NET) formation, degranulation, and the production of reactive oxygen species (ROS) (18–21). Further to this, neutrophils can release various pro-inflammatory cytokines (22–24). Murine studies show that neutrophils egress from the bone marrow and into the circulation having already pre-synthesised their ammunition and as such do not require much transcriptional or translational input to respond to an invading microbe (25), thereby allowing them to rapidly utilise their effector mechanisms. As such, neutrophils have evolved to be an excellent first line of defence against invading microbes.

Despite evolving to effectively kill, whether neutrophils are capable of directly killing Mtb is a contentious issue. Evidence suggests that neutrophils can kill Mtb, however not all data recapitulates these results (26, 27). One of the primary effector mechanisms utilised by neutrophils to combat Mtb is phagocytosis (28, 29). Neutrophils ingest microbes through the formation of membrane protrusions which engulf the target microbe. Once internalised, phagosome maturation occurs, enabling granules within the neutrophil to fuse with the phagosome membrane and deliver soluble or membrane-bound effector proteins into the phagosome to kill the entrapped microbe (30–32). Neutrophils can phagocytose Mtb, however whether they can kill the ingested bacteria remains uncertain. One study showed that neutrophils from healthy individuals were able to phagocytose Mtb and rapidly kill Mtb within an hour, a process which was enhanced by treating neutrophils with TNF-α (28), however other studies contradict these results, observing no killing of Mtb following phagocytosis of the bacteria (27).

Neutrophils also possess opsonic receptors, which recognise host-derived proteins bound to target pathogens. These proteins, known as opsonins, are used to identify invading pathogens and label them as a target for elimination through processes such as antibody-dependent phagocytosis (ADP) or ADCC. Neutrophils can also recognise opsonised targets and degranulate to release potent antimicrobial agents into the extracellular space surrounding the target microbe (33, 34). Opsonisation of Mtb with fresh human serum was shown to increase the ability of human neutrophils to phagocytose the bacteria (27). Following phagocytosis, ROS can also be generated to eliminate entrapped microbes within the phagosome (35). NADPH oxidase (NOX) allows for the generation of superoxide within the phagosome (36). The importance of ROS in host defence is clearly illustrated in patients with chronic granulomatous disease (CGD). CGD is a rare, inherited disease which results in neutrophils which are incapable of producing ROS due to a dysfunction with their NOX. Consequently, patients with CGD are hypersusceptible to various bacterial and fungal infections (37). Remarkably, neutrophils from patients with CGD were shown to be no less capable of killing Mtb compared with normal neutrophils, suggesting that neutrophils do not utilize ROS to kill Mtb (38). This was further confirmed by a paper showing that the use of ROS inhibitors did not affect the ability of neutrophils to kill Mtb (28). Interestingly, another study showed that despite the prompt production of ROS in neutrophils following Mtb infection, Mtb can survive within neutrophils. This persistence was followed by necrotic cell death of Mtb infected neutrophils, which was shown to be dependent on ROS, as neutrophils from patients with CGD were protected from this Mtb-induced necrosis (27). This suggests that having CGD could potentially benefit neutrophils during the acute stage of Mtb infection.

Another weapon employed by neutrophils during infection is the release of NETs. When a neutrophil becomes activated, it can modify its own chromatin and release it along with granule proteins into the tissue to bind to bacteria, fungi and viruses through a process of cell death known as NETosis (39, 40). Mtb infection of human neutrophils *in vitro* induces ROS production and NET formation, however, neither the neutrophil-induced ROS nor their NETs are able to kill Mtb (41). While the presence of NETs may be beneficial to contain infection, they may also be deleterious to the host, as they may shield Mtb and block immune cell recruitment, leading to an impaired clearance of infection, while also further disseminating inflammation (41).

Human neutrophils also produce numerous different pro- and anti-inflammatory cytokines upon stimulation, such as TNF-α, IL-1β, IL-6 and IFN-γ, as well as a wide array of chemokines to recruit other immune cells to the site of infection (22–24, 42). TNF is an essential cytokine for the host response to infection. The essential role of TNF-α in Mtb infection is clearly seen in patients on anti-TNF-α therapies, who are much more likely to develop Mtb (43). TNF-α can be both protective and pathogenic during TB, where it can induce ROS in infected macrophages, which initially increases anti-microbial activity but can then lead to programmed necrosis of cells (44). The production of IL-1β promotes cytokine production, such as that of TNF-α and IL-6, from bystander cells such as other neutrophils and macrophages (45, 46). It promotes the polarisation of Th17 cells which are important adaptive mediators of infectious disease, and it augments bacillary killing of Mtb (47, 48). IL-6 is a pleiotropic cytokine which is an essential mediator of both innate and adaptive immunity (49). Murine studies show that knocking out IL-6 makes mice more susceptible to infection with an array of bacteria, viruses, fungi and parasites (50). Neutrophils have also been shown to produce IFN-γ in response to *Streptococcus pneumoniae* and as such are an essential early source of IFN-γ, which plays an essential role in bacterial clearance (24, 51). The effect of these cytokines on neutrophil survival and function will be discussed in the next section. Taken together, these studies illustrate a key role for neutrophil-mediated immunity against Mtb infection in addition to neutrophil-mediated damage in TB disease. Given the complexity of Mtb infection and TB disease, it is unsurprising that conflicting evidence exists as to the role of neutrophils in protection versus pathogenesis. Examining the changing role of neutrophils across disease progression using models of early clearance and end stage disease will help to delineate this further.

## Neutrophil interactions with other immune cells

### Macrophages and monocytes modulate neutrophil recruitment and effector functions

**Figure 2:**
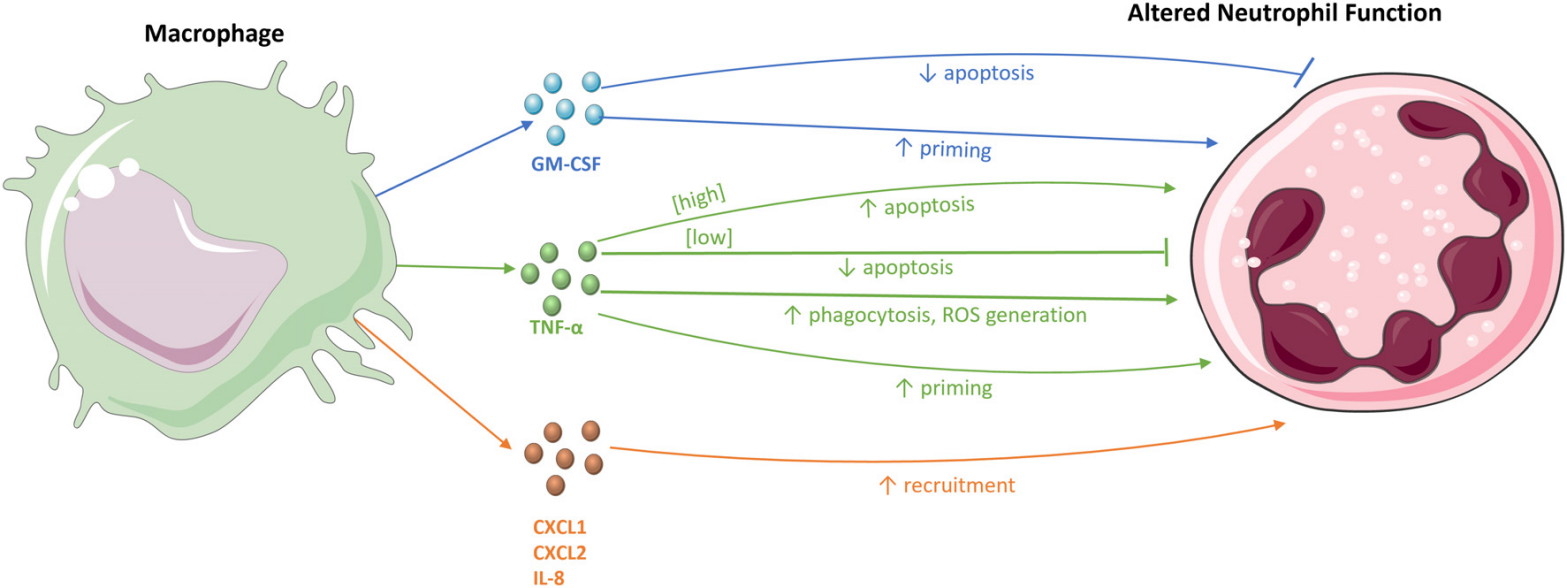
The effects of macrophages on neutrophils. Macrophages and their chemokines, CXCL1, CXCL2, and IL-8, recruit neutrophils to sites of infection and inflammation. The macrophage-derived cytokines, TNF-α and GM-CSF, modulate neutrophil survival, either by inhibiting or stimulating apoptosis. These cytokines also influence various immune effector functions of neutrophils, for example, by priming the respiratory burst and increasing neutrophil phagocytosis and ROS generation.

Many studies have shown the interaction between macrophages and neutrophils (**Figure 2**). Macrophages can produce numerous mediators to recruit neutrophils to a site of infection. For example, intraperitoneal LPS stimulation in mice to induce peritonitis results in the production of the neutrophil chemoattractants CXCL1 and CXCL2 by tissue macrophages (5, 52). Blocking the activity of CXCL1 and CXCL2 significantly reduces neutrophil infiltration into the peritoneal cavity (52), as does antagonism of their receptor, CXCR2 (53). Moreover, macrophage depletion in mouse peritonitis models leads to the inhibition of neutrophil migration to the peritoneal cavity. Despite some neutrophil infiltration into the peritoneal wall, the neutrophils were incapable of penetrating further into the cavity, emphasising the role of macrophages in the deep migration of neutrophils into tissues (52).

IL-8 (CXCL-8) is a potent activator and chemoattractant of neutrophils that acts on the human CXCR1 and CXCR2 receptors. While mice do not have a homologous IL-8 gene, murine CXCR is similar to human CXCR2 and binds to various IL-8-like CXC chemokines, such as CXCL1 and CXCL2 (54). CLEC9A is a C-type lectin receptor expressed on macrophages, the ligation of which drives IL-1β and IL-8 production by macrophages. Knockdown of CLEC9A in mice during Mtb infection results in reduced expression of both IL-1β and IL-8, resulting in decreased neutrophil migration, demonstrating the importance of CLEC9A interaction with Mtb for macrophage-mediated neutrophil recruitment in Mtb infection (55).

In a murine model of glomerulonephritis in which glomerular injury is highly neutrophil-dependent, monocyte depletion significantly reduces neutrophil recruitment to glomerular capillaries (56). However, given that monocytes differentiate into macrophages, it is possible that the depletion of monocytes causes a reduction in macrophage populations, which then results in fewer neutrophils being recruited. Further studies will be required to distinguish between the direct effects of monocytes on neutrophils and those mediated indirectly through macrophages. Neutrophils that did not engage in contact with monocytes or the monocyte-derived chemokines CXCL1 and CXCL2 spent a reduced amount of time in glomerular capillaries, highlighting the role of monocytes in neutrophil retention at sites of inflammation (56). The cooperation between specific immune cells and neutrophils can also be key for pathogen clearance as highlighted by previous studies. For example, primary human macrophages can acquire neutrophil granules for antimicrobial activity against intracellular pathogens, such as Mtb and BCG (57). Efferocytosis of apoptotic neutrophils has also been shown to enhance Mtb clearance in HIV-coinfected human macrophages, through the actions of myeloperoxidase (58). Furthermore, extracellular vesicles secreted from Mtb-infected neutrophils can also promote macrophage bactericidal activity (59). Therefore, the cooperation between many of these immune cells and neutrophils, and the mechanisms they employ to kill invading pathogens, should not be neglected.

Exposure to pro-inflammatory cytokines also delays apoptosis, extending neutrophil lifespan, which allows sufficient time for neutrophils to aid in host defence (3). For example, incubating human neutrophils with GM-CSF and IL-1β *in vitro* delays neutrophil apoptosis (3). This effect is mediated by inducing the expression of the protein myeloid cell leukaemia-1 (Mcl-1), a member of the B cell lymphoma-2 (Bcl-2) family of proteins which has anti-apoptotic effects through the inhibition of the pro-apoptotic protein Bcl-2-associated-X (Bax). Both GM-CSF and IL-1β are produced by macrophages and help to maintain sufficient levels of Mcl-1 expression to counteract Bax (3). GM-CSF also suppresses apoptosis by inhibiting the cleavage of pro-caspase-3 into active caspase-3 in neutrophils, reducing caspase-3-mediated apoptosis (60, 61). Interestingly, a clinical trial involving AIDS patients also found that infusion with GM-CSF augmented phagocytosis and intracellular killing of *S. aureus* by neutrophils (62).

TNF-α, another macrophage-derived cytokine, has dual regulatory actions on neutrophil apoptosis, with differential effects that are concentration-dependent and operate through different mechanisms. Low TNF-α concentrations delay apoptosis, while high concentrations initiate apoptosis. Incubation of human neutrophils with 10 ng/mL of TNF-α *in vitro* results in elevated levels of apoptosis due to increased turnover of Mcl-1 in a caspase-dependent manner (63). However, 1 ng/mL of TNF-α induces increased levels of the anti-apoptotic Bcl-2-related protein, protecting neutrophils from apoptosis. In this way, macrophages may exert opposing effects on the rate of neutrophil apoptosis. While the prolonged survival of neutrophils is important early in infection to aid in the clearance of pathogens, their subsequent inhibition is equally crucial to resolve inflammation and reduce tissue damage commonly caused by neutrophils (7). In addition to regulating survival, macrophages can enhance neutrophil effector functions through cytokine stimulation. The culture of human neutrophils with Mtb shows that the ability of neutrophils to phagocytose Mtb is significantly increased upon TNF-α stimulation (28). TNF-α also enhances the assembly of NOX and the subsequent activation of the respiratory burst. *Ex vivo* studies show that pre-treatment of human neutrophils with TNF-α prior to infection enhances the killing of *S. aureus* (64).

### Dendritic cells promote neutrophil infiltration and survival at sites of infection

Like macrophages and monocytes, Dendritic cells are important in the regulation of neutrophil recruitment and survival in sites of infection. Dendritic cells both promote neutrophil infiltration to aid in fighting infection and later limit their recruitment to prevent excess tissue damage. In a murine model of *Propionibacterium acnes* (*P. acnes*) infection in which intradermal injection of *P. acnes* leads to high levels of neutrophil infiltration at sites of infection, the depletion of type I conventional dendritic cells (cDC1s) results in reduced inflammation due to the decreased infiltration of neutrophils and other inflammatory cells (65). Additionally, depletion of cDC1s compromises the ability of neutrophils to produce NETs and increases neutrophil apoptosis. This is due to the decreased expression of genes that typically inhibit apoptosis and the upregulated expression of pro-apoptotic Bcl-2 proteins. Furthermore, cDC1-derived VEGF has been shown to mediate neutrophil recruitment following infection with *E. coli* in both mouse and human models of infection (65). Conversely, cDC1s can also inhibit neutrophil recruitment through the actions of CLEC9A. CLEC9A senses tissue damage by binding F-actin exposed by necrotic cells. CLEC9A deficiency in a mouse model of acute pancreatitis leads to increased morbidity and mortality (66). Neutrophil infiltration is enhanced in the absence of CLEC9A, demonstrating the importance of CLEC9A in reducing neutrophil infiltration (66).

### The crucial role of T cells and their effector cytokines in the regulation of neutrophil defence and pathology

Neutrophils and T cells are present and interrelate in the lymph nodes and tissues in both health and disease (67, 68). Neutrophils and T cell interactions represent a spectrum of states with profound effects on T cell phenotype and function, depending on the inflammatory milieu and subtle variations in neutrophil populations or T cell subsets (69). T cells modulate neutrophil activation and function through the recruitment of neutrophils to the site of infection and by delaying neutrophil apoptosis (70, 71). CD4+ T cells play a critical role in Mtb infection due to their dynamic crosstalk interactions with neutrophils. Among the T helper cell populations, Th17 and Th1 cells are the primary mediators of protection and pathology in TB disease owing to their relationship with neutrophils.

Th17 cells play an important role in the stimulation and enlistment of neutrophils into the site of infection as well as activating the differentiation of granulopoietic lineage cells and promoting inflammation (72). Th17 cells stimulate neutrophil activities directly through the release of IL-8 (73). Th17 effector cytokine IL-17 acts indirectly by stimulating epithelial cells to produce neutrophil chemoattractants, such as IL-8, CXCL1 and GM-CSF. Secretion of these cytokines by human epithelial cells was found to be enhanced *in vitro* through co-culture with IL-17 and TNF-α, indicating a role for Th17 cells in recruiting neutrophils (74). IL-17 also reduces the GM-CSF-induced increase in Mcl-1 levels and attenuates the inhibitory effects of Mcl-1 on Bax and impairs GM-CSF inhibition of caspase-3 (60). Therefore, IL-17 does not directly induce neutrophil apoptosis, rather it counteracts the anti-apoptotic effects of GM-CSF. It has also been reported that Th17 lineage associated cytokines IL-17 and IL-23 can alter the phenotype of neutrophils, skewing them towards mediating severe tissue inflammation pathology by triggering the secretion of matrix metalloproteinases associated with tissue destruction and remodelling such as MMP-9 and the release of myeloperoxidase granules MPO in the lung (75). One study identified that in lung biopsies of human pulmonary TB patients, BAL fluid, and in primary human airway epithelial cells, IL-17 regulates MMP secretion (76). Moreover, repeated BCG vaccination in Mtb-infected mice caused elevated IL-17, TNF-α, IL-6, and MIP-2, and encouraged the infiltration of neutrophils into the lungs, mediating damage (77). There is also evidence to suggest that the interactions between Th17 cells and neutrophils are protective during acute infection and damaging in chronic infection (72). TB pathology is highly associated with the degree of inflammation and Th17 cell-mediated neutrophil recruitment to the site of infection can result in excessive tissue damage in redundancy neutrophils, severe inflammation, bad prognosis, and lung pathology (78).

Th1 cell-derived IFN-γ also exhibits many effects on neutrophil function. The expression of various cytokines and chemokines by neutrophils is modulated by IFN-γ. Chemokines involved in neutrophil recruitment, such as IL-8 and CXCL1, are downregulated, while chemokines which function to recruit cells of the adaptive immune system are upregulated, for example IP-10, a chemoattractant for T cells (79). Thus, IFN-γ may signal to reduce neutrophil infiltration and promote the transition to adaptive immune responses (79). Additionally, IFN-γ regulates the expression of various surface markers on neutrophils. IFN-γ upregulates the expression of receptors and integrins involved in neutrophil recruitment, such as CD11b and CD18, in addition to those which aid in activation, such as CD14 which binds LPS (79). Interestingly, neutrophil expression of the IFN-γ receptor is rapidly downregulated upon IFN-γ stimulation, suggesting a potential control mechanism to limit excess neutrophil-mediated tissue damage (80).

IFN-γ also induces priming of neutrophils by enhancing the respiratory burst when combined with a second stimulus, such as N-Formylmethionine-leucyl-phenylalanine (fMLP) (4, 79). While many other priming agents carry out their actions through the phosphorylation and upregulation of NOX subunit p47phox (4), IFN-γ upregulates the expression of gp91phox, a membranal component of NOX (79). IFN-γ is also capable of enhancing neutrophil phagocytosis through the upregulation of ROS, leading to increased antimicrobial activity. Additionally, neutrophil ADCC is augmented following IFN-γ stimulation, which is associated with elevated expression of the high-affinity IgG receptor, FcγRI. FcγRI ligation triggers increased microbicidal activity and oxidative burst through NOX activation (79, 81). Furthermore, like the macrophage-derived cytokines TNF-α and GM-CSF, IFN-γ prolongs neutrophil survival by suppressing apoptosis. This demonstrates the opposing regulatory effects of the Th17 cell-derived cytokine IL-17, which induces apoptosis, and Th1 cell-derived IFN-γ, which prolongs neutrophil survival (3, 63, 79).

While neutrophils are not classically viewed as antigen-presenting cells, they have been shown to express major histocompatibility complex (MHC) class II but not the co-stimulatory molecules CD80 and CD86 in their resting state. Co-incubation with CD4+ T cells can upregulate MHC class II and CD80/86 expression on neutrophils, however. This elevated expression is facilitated by the cytokines IFN-γ and TNF-α and direct cell-to-cell contact between neutrophils and T cells. Neutrophils expressing these molecules are capable of antigen presentation to T cells and induce T helper cell differentiation (79, 82, 83). Given the aforementioned effects of T cells on neutrophils, these findings potentially illustrate a positive feedback loop in which T cell-activated neutrophils provide stimulation to T cells, which then further enhance neutrophil activity (82).

CD8+ T cells modulate neutrophil survival and alter the expression of various surface markers *via* the production of the cytokines IFN-γ, TNF-α, and GM-CSF. In both co-culture and transwell experiments, anti-CD3-activated human CD8+ T cells induced the expression of the activation markers CD11b, CD64, and CD62L on the surface of neutrophils (71). Additionally, neutrophils cultured with activated CD8+ T cells displayed a significantly reduced rate of apoptosis compared to those cultured alone (71, 84). Interestingly, these results were reproduced when neutrophils were cultured with the supernatants from the CD8+ T cells which contained the cytokines IFN-γ, TNF-α, and GM-CSF, the effect of which was abrogated using neutralising antibodies (71). Much like Th1 cells, CD8+ cell-derived IFN-γ is also capable of inducing the expression of MHC class II molecules on the surface of neutrophils (84).

### B cells induce neutrophil MHC II expression and reduce neutrophil infiltration

While many immune cells are vital in neutrophil recruitment, B cells regulate this response to prevent excessive neutrophilia by interfering with neutrophil motility, thereby slowing their recruitment to sites of infection (85). In B cell-deficient mice infected with Mtb, neutrophil infiltration and inflammation in the lungs was increased, demonstrating a role for B cells in reducing neutrophil recruitment in Mtb infection (85, 86). In a study examining the effects of BCG vaccination in B cell-deficient mice, it was shown that the inhibitory effects of B cells on neutrophil migration are likely due to active suppression of CXCL1, CXCL2, and G-CSF (85). Physical interaction between B cells and neutrophils takes place in the lungs in a β2 integrin (CD18)-dependent manner. As aforementioned, MHC class II molecules are not usually expressed by neutrophils, however neutrophils can acquire MHC class II from B cells through direct cell-to-cell contact (87). B cells induce apoptosis in aged CXCR4+ neutrophils through increased caspase-3 activation. Experimental depletion of B cells in mice also allows neutrophils to persist in the lungs and propagate inflammation, resulting in the development of fibrotic interstitial lung disease (87). Thus, the effects of B cells on neutrophils are centred around controlling and limiting their inflammatory effects, both through preventing their migration to tissues and initiating apoptosis.

### NK cells have opposing effects on neutrophil survival

Natural killer (NK) cell-mediated effects on neutrophils are focused on the regulation of apoptosis. Co-culture of neutrophils with supernatants from IL-2-activated human NK cells inhibits neutrophil apoptosis *in vitro* (88). Upregulation of CD11b and loss of CD62L are common surrogate markers of neutrophil activation. In the presence of the supernatant from cytokine-activated NK cells, CD11b expression increases, however, CD62L levels do not change. Additionally, neutrophils cultured with activated NK cell supernatant display enhanced phagocytic activity and ROS production. These effects are mediated by soluble factors released by NK cells in response to activation, like TNF-α, IFN-γ, and GM-CSF (88). Conversely, direct contact between NK cells and neutrophils promotes neutrophil apoptosis in a caspase-dependent manner. This effect can overcome the pro-survival mechanisms of the aforementioned cytokines *in vitro*. There is a fine balance between the pro-apoptotic and pro-survival actions of NK cells on neutrophils. Following activation, human neutrophils rapidly decrease the expression of human leukocyte antigen class I (HLA-I) molecules both *in vitro* and *in vivo*. As these molecules are ligands of inhibitory NK cell receptors, their downregulation causes decreased inhibitory signalling. This allows the action of activating NK cell receptors to dominate, resulting in the exertion of cytotoxic effects of NK cells on neutrophils (89). Overall, NK cells have divergent effects on neutrophil survival depending on the balance of cell contact dependent signaling and soluble mediators.

**Figure 3:**
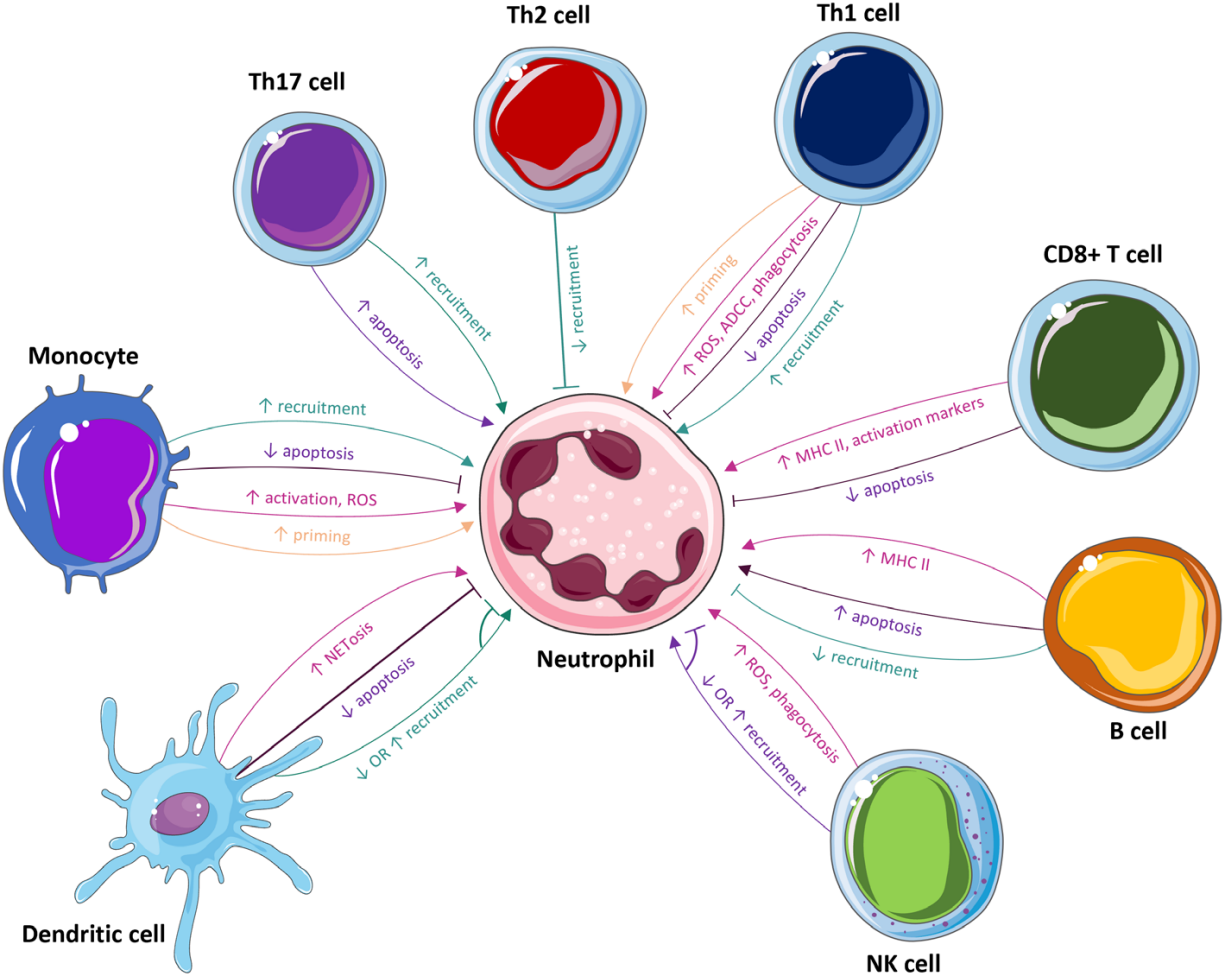
The influence of immune cells on neutrophils. Many immune cells, including monocytes, Th17 cells, Th2 cells, Th1 cells, CD8+ T cells, B cells, NK cells, dendritic cells, and mast cells, modulate neutrophils. These cells affect neutrophil recruitment, priming, survival, activation, and effector functions, such as ROS production, NETosis, phagocytosis and ADCC.

In summary, while the mobilisation of neutrophils is vital in the initial stage of infection, their actions must be tightly regulated to prevent excessive tissue destruction that is potentially fatal to the host. Accordingly, different immune cells both recruit and activate neutrophils during acute infection and then proceed to inhibit their effector functions and induce apoptosis to prevent excess inflammation and facilitate a return to homeostasis. It’s also important to note that neutrophil responses could also vary depending on the neutrophil subtype in question (90, 91).Thus, understanding how immune cells interact with neutrophils could permit more control over their effector functions through potential therapeutic intervention (**Figure 3**).

## Metabolic pathways involved in neutrophil effector functions and their modulation in response to pathogen stimulation

**Figure 4:**
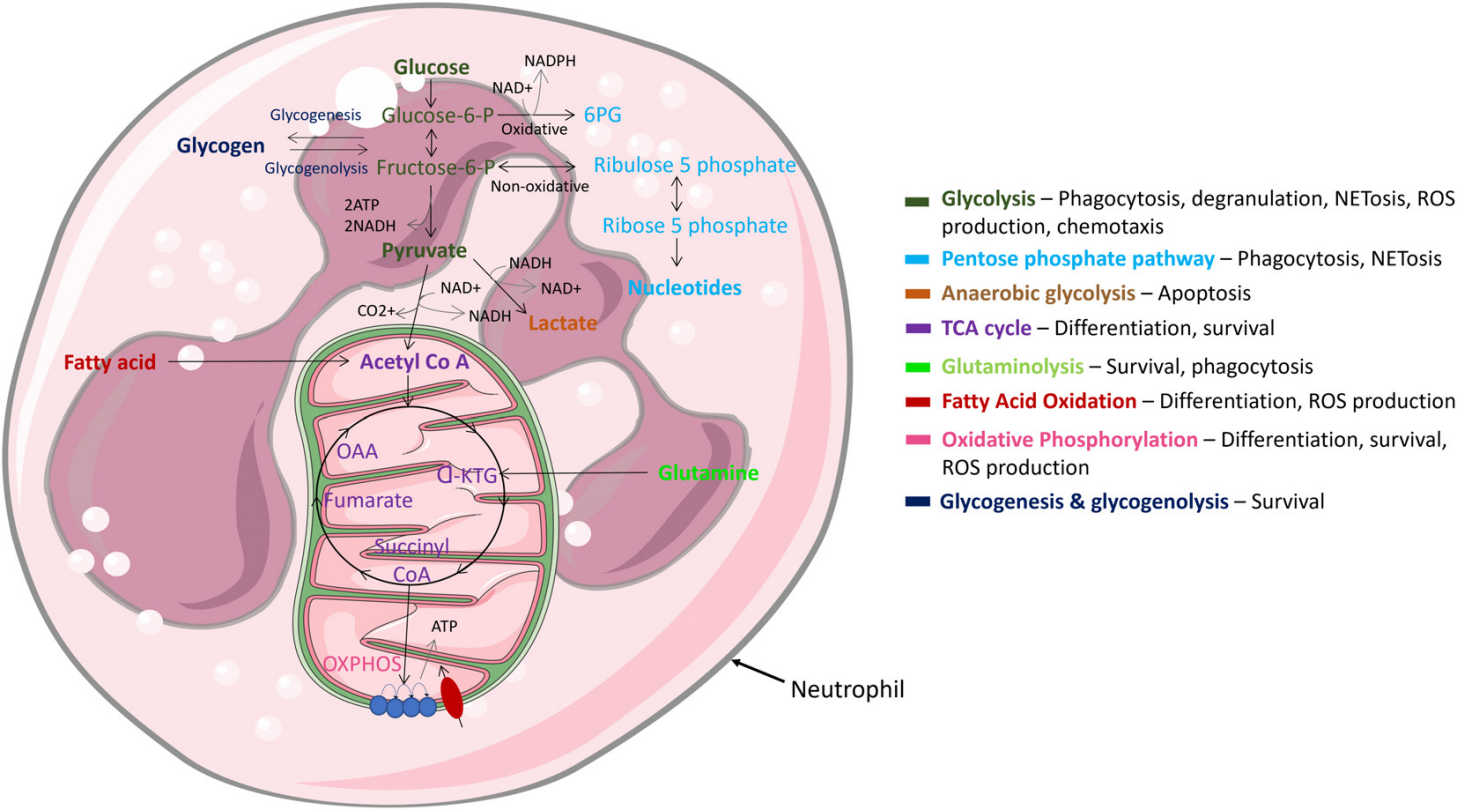
Main metabolic pathways in neutrophils. Neutrophils are highly dynamic cells which adapt different metabolic pathways to carry out specific effector functions. Glycolysis, glycogen and FAO metabolism are extensively used, while mitochondrial metabolism is rarely utilised and mainly functions to regulate cell survival & apoptosis.

Being highly dynamic cells, neutrophils utilise diverse metabolic pathways for growth, proliferation, survival, and their death (**Figure 4**). As nutrient availability fluctuates in their microenvironment, neutrophils can differentially rewire their metabolic pathways to adapt to new environmental demands. Like other cytotoxic cells, neutrophils require a large amount of energy to perform their effector functions and have specialised metabolic requirements for different functions (92–95). The cells rely on glycolysis as a dominant source of ATP; accordingly, neutrophils contain very few mitochondria and employ low tricarboxylic acid (TCA) cycle and oxidative phosphorylation (OXPHOS) rates (95). Neutrophil metabolic plasticity enables them to function and survive in harsh microenvironments, such as hypoxia (96). Moreover, increased succinate production in individuals with a mutation in succinate dehydrogenase enhances neutrophil survival, confirming a role of the TCA cycle in regulating neutrophil survival (97). Glycolysis is considered the most fundamental metabolic pathway linked to optimal neutrophil function. Importantly, various studies have shown that when glucose is depleted, effector functions of neutrophils are significantly abrogated (92, 93, 98, 99). While it is evident that glycolysis is key for neutrophil effector function, recent studies have called into question the long-held belief that they are solely dependent on glycolysis. For example, neutrophils derived from diabetic rat models with impaired glucose and glutamine metabolism activate compensatory FAO metabolism. Additionally, fatty acid synthesis (FAS) is utilised to build neutrophil membranes during differentiation and to replenish membranes post exocytosis (100). Neutrophils have also evolved to utilise multiple metabolic substrates to generate energy stores in the form of glycogen, which are dynamically regulated by both gluconeogenesis and glycogenesis (101). However, this is mainly important in driving effector functions during infection, not homeostasis.

### During activation and infection, bioenergetics underpins the neutrophils’ response to infection

Neutrophil effector functions during inflammation are strongly linked to the metabolic state of the cell. Metabolic rewiring also occurs during activation and infection, to adapt to the infection and produce enough energy to fulfil its host defensive duties. An increase in glucose metabolism is a typical side effect of activation and infection in neutrophils (102, 103). NET release, for example, is controlled by glucose concentration (104). Neutrophil mediated phagocytosis has also been shown to utilise glucose metabolism (105). Hexokinase, a key enzyme which converts glucose to glucose-6-phosphate, translocates from the cytosol towards the site of its phagocytic target, binding in response to stimulation with fMLP, as well as other neutrophil activating stimuli (106).

Neutrophils do not solely rely on glycolysis during the immune response to pathogens. Treating neutrophils with the glycolytic inhibitor, 2DG, and the OXPHOS inhibitor, dimethyl malonate (DMM), significantly inhibits TNF-α and ROS production in a murine model of *L. monocytogenes* infection, implicating both pathways in anti-microbial activity (107). A study also showed that neutrophils require the production of intracellular glycogen reserves *via* gluconeogenesis and glycogenesis upon activation (101). Interestingly, peripheral blood-derived neutrophils isolated from COPD patients are unable to regulate glycogen synthesis, resulting in diminished intracellular glycogen stores, and consequently, defective killing of *S. aureus* and reduced survival (102). Therefore, glycogen metabolism facilities microbial killing by keeping the neutrophil alive long enough to eliminate its target.

During viral infections, neutrophils heavily catabolise glucose. For example, neutrophils isolated from individuals experiencing SARS-CoV-2 induced pneumonia display dysfunctional mitochondria, glycogen build-up in the cytoplasm, and enhanced glycolysis and glycogenolysis (108). Considering the recent revelations of the changes in the pathophysiology of neutrophils in COVID-19 infections, and their highly elevated presence in infected lungs (109), investigations into alterations in neutrophil metabolism in response to SARS-CoV-2 may be necessary to facilitate future COVID-19 treatment strategies. For example, treatment of individuals experiencing severe COVID-19 with dexamethasone, a glucocorticoid used to treat inflammatory conditions, significantly improves mortality. Specifically, dexamethasone downregulates the expression of interferon stimulated genes and promotes the expansion of immunosuppressive neutrophils (110). A major part of the drug’s efficacy in treating COVID-19 thus comes from its ability to suppress detrimental neutrophil activity (110).

ROS production is a core instrument of neutrophil activation and is necessary to effectively eliminate microbes. During respiratory burst, NOX utilises oxygen and NADPH to generate O_2_-. NOX activation necessitates the transfer of some of its cytoplasmic components such as p47phox to the membrane (111). At the membrane, _phox_, p47_phox_ binds to gp91_phox_ and p22_phox_, is phosphorylated and in its activated state, catalyses the conversion of oxygen to ROS. This process is impaired in patients with CGD, and their granulocytes are unable to kill certain pathogens. While this translocation is generally a mark of glycolytic metabolism, NOX-dependant oxygen consumption is dependent on the pentose phosphate pathway (PPP). In the cytosol, the PPP pathway employs the glycolytic intermediate glucose-6-phosphate to create ribose-5-phosphate and NADPH. This NADPH is then used as a NOX substrate (112).

### The importance of neutrophil metabolism during Mtb infection

The phagocytosis of Mtb by neutrophils has been shown to be independent on the activation state of the neutrophil. Neutrophils which phagocytose large amounts of fatty acids, LDLs and oxidised LDL, are more permissive to infection, since they are a carbon source to feed Mtb (113). Another study also elucidated the importance of glutamine metabolism during Mtb infection of neutrophils (114). Mtb has been shown to promote neutrophil apoptosis to aid immune escape. As glutamine has been proven to delay neutrophil apoptosis, glutaminolysis may be necessary to counteract this effect (115). Indeed, adding glutamine to neutrophil cultures has been shown to increase their phagocytic capabilities and ROS production, both important mechanisms in pathogen elimination (116). While this gives insight into another method glutamine metabolism may contribute to anti-Mtb activity, more research into the precise involvement of glutaminolysis in Mtb activated neutrophils is needed. Evidence also suggests that neutrophil NET formation in Mtb-activated neutrophils is dependent on the phagocytosis of Mtb (117). All primary neutrophil effector functions employed in response to Mtb infection, namely NETosis, ROS production and phagocytosis, are highly glycolytic-dependent processes (118). This reiterates the importance of glycolysis for anti-Mtb activity. Precisely how Mtb impacts neutrophil immunometabolism in the lung and how this underpins neutrophil effector functions has yet to be examined. Moreover, the effect of other infected lung immune cells on bystander neutrophil function should also be examined to evaluate what extent the lung microenvironment signals and influences neutrophil biology.

## Therapeutically targeting host neutrophil metabolism and function

Current anti-microbial drugs directly target the invading pathogen. Recent insights into understanding cell biology are leading to the identification and development of a wide range of HDTs. HDTs work by interfering with host cell components required for pathogen replication and persistence, enhancing protective immune responses against pathogens, reducing severe inflammation, and balancing immunological reactivity at infection sites. This emerging approach aims to support the function of infected host cells along with other therapies. If neutrophils could be targeted through disease-specific mechanisms without interrupting their major immune and homeostatic functions, such interventions could hold significant promise in the treatment of various inflammatory and infectious diseases (119).

Neutrophil infiltration is undoubtedly a defining hallmark of severe infectious disease, including during Mtb and SARS-CoV-2 infection. Manipulating neutrophil activity in several pulmonary diseases characterised by excessive neutrophil-mediated tissue damage is an area of intense research. HDTs could be targeted towards the modulation of activation or the inhibition of neutrophil responses since many clinical conditions are associated with either hyper- or hypoactivation of the neutrophil. These agents could also target survival and/or apoptosis in neutrophils (120), for example, targeting the pro-survival factor Mcl-1 and/or PI-3 kinases (121).

Given the conflicting roles of neutrophils in TB, neutrophils represent a compelling target of HDTs, particularly when their role in the context of TB and other diseases becomes clearer (13, 122). Iron chelators exhibit opposing functional effects on neutrophils, with some activating and some inhibiting neutrophil effector functions. For example, the use of iron chelators, such as desferrioxamine, enhances the immune function of human macrophages infected with Mtb and promotes glycolysis, demonstrating it as a possible HDT during early Mtb infection (122). Indeed, desferrioxamine has been shown to enhance NET formation by stimulating ROS production *in vitro*, a potent antimicrobial mechanism in neutrophils (123). Conversely, another study demonstrates that the iron chelator deferasirox inhibits both ROS and NET formation, an effect which could be therapeutically beneficial in abrogating unwanted inflammatory responses (124). However, given the conflicting evidence surrounding the benefit of NET formation during Mtb infection, it is debatable whether targeting NETosis is a viable HDT option during the early stages of Mtb infection. Therefore, the specific involvement of neutrophils in TB should be fully elucidated before HDTs targeting neutrophils can be deployed. Targeting the glycolytic enzyme phosphofructokinase-1 liver type could also be beneficial too, as previously described using the small molecular inhibitor, NA-11 (125). Accordingly, altering the fate of glucose by modulating this key enzymatic step could dramatically alter the function and fate of neutrophils, to strategically target their immunometabolic and functional processes (119).

Other components have also been investigated as potential neutrophil targets of HDTs for TB. For example, targeting ROS production and/or the ESX-1 pathway could hold some merit in reversing Mtb-induced necrosis of human neutrophils (126). Another study showed that BCG vaccination causes functional changes in human neutrophils, increasing antimicrobial activity against unrelated pathogens, where the authors suggest that trained immunity may be a therapeutic target to modulate neutrophil effector function (127). Enhancing neutrophil function during early Mtb infection may be beneficial, but detrimental in later disease. Thus, further investigation into the therapeutic targeting of neutrophils during early and late Mtb infection is thus required.

## Concluding remarks

Neutrophils play both an essential role in host defence against invading microorganisms and are involved in the pathogenesis of several inflammatory diseases. In Mtb infection, they provide a crucial first line of defence. Neutrophils interact with and regulate the function of many other immune cells, including various T cell subsets, monocytes and macrophages, B cells and NK cells. Neutrophil function and their effector responses are also defined by the microenvironment they find themselves in. For example, exposure to cytokines such as TNF-α, IL-1β, IFN-γ and GM-CSF, can drive neutrophil activation as well as amplify neutrophil recruitment to the site of infection. Moreover, the current review did not discuss how immune cells in the lung interact with diverse subsets of neutrophils. Accordingly, to put into context the complexity of the interactions between immune cells and neutrophils in the lung, how neutrophil diversity and heterogeneity also effects the lung microenvironment in the setting of infection must also be investigated in future studies (90, 91, 128). Understanding how neutrophils are activated, their crosstalk with other cells and how they become dysregulated could help to direct the development of therapeutic strategies to maintain the crucial balance between their beneficial and detrimental effects.

## Author contributions

PJ: conceptualization and software. PJ, KJ and BS: funding acquisition and administration. PJ, GE, MD, WA and CS: visualization and writing-original draft. All authors: validation, writing-review, and editing. All authors contributed to the article and approved the submitted version.

## Funding

This work was supported by the Irish Research Council (GOIPD/2020/16), the Health Research Board (EIA-2019-010) and by The Royal City of Dublin Hospital Trust (Baggot Street Hospital, Dublin 4, Ireland).

## Conflict of interest

The authors declare that the research was conducted in the absence of any commercial or financial relationships that could be construed as a potential conflict of interest.

## Publisher’s note

All claims expressed in this article are solely those of the authors and do not necessarily represent those of their affiliated organizations, or those of the publisher, the editors and the reviewers. Any product that may be evaluated in this article, or claim that may be made by its manufacturer, is not guaranteed or endorsed by the publisher.
